# VIVALDI ASCOT and Ethnography Study: protocol for a mixed-methods longitudinal study to evaluate the impact of COVID-19 and other respiratory infection outbreaks on care home residents’ quality of life and psychosocial well-being

**DOI:** 10.1136/bmjopen-2024-088685

**Published:** 2024-08-07

**Authors:** Lavinia Bertini, Nicola Schmidt-Renfree, James Blackstone, Oliver Stirrup, Natalie Adams, Iona Cullen-Stephenson, Maria Krutikov, Ruth Leiser, Lara Goscé, Catherine Henderson, Paul Flowers, Laura Shallcross, Jackie A Cassell, Dorina Cadar

**Affiliations:** 1 Department of Clinical Neuroscience, Brighton and Sussex Medical School, Brighton, UK; 2 Department of Primary Care, Brighton and Sussex Medical School, Brighton, UK; 3 Comprehensive Clinical Trials Unit, University College London, London, UK; 4 Institute for Global Health, University College London, London, UK; 5 Royal Free London NHS Foundation Trust, London, UK; 6 UCL Institute of Health Informatics, London, UK; 7 Institute of Health informatics, University College London, London, UK; 8 Department of Psychological Sciences and Health, University of Strathclyde, Glasgow, UK; 9 Department of Infectious Disease Epidemiology, London School of Hygiene & Tropical Medicine, London, UK; 10 Care Policy and Evaluation Centre, The London School of Economics and Political Science, London, UK; 11 University of Strathclyde, Glasgow, UK; 12 UK Health Security Agency, London, UK; 13 Behavioural Scince and Health, University College London, London, UK

**Keywords:** COVID-19, Quality of Life, Depression & mood disorders, Social Support, Respiratory infections, Quality in health care

## Abstract

**Abstract:**

**Introduction:**

Older adults in care homes experienced some of the highest rates of mortality from SARS-CoV-2 globally and were subjected to strict and lengthy non-pharmaceutical interventions, which severely impacted their daily lives. The VIVALDI ASCOT and Ethnography Study aims to assess the impact of respiratory outbreaks on care home residents’ quality of life, psychological well-being, loneliness, functional ability and use of space. This study is linked to the VIVALDI-CT, a randomised controlled trial of staff’s asymptomatic testing and sickness payment support in care homes (ISRCTN13296529).

**Methods and analysis:**

This is a mixed-methods, longitudinal study of care home residents (65+) in Southeast England. Group 1—exposed includes residents from care homes with a recent COVID-19 or other respiratory infection outbreak. Group 2—non-exposed includes residents from care homes without a recent outbreak. The study has two components: (a) a mixed-methods longitudinal face-to-face interviews with 100 residents (n=50 from group 1 and n=50 from group 2) to assess the impact of outbreaks on residents’ quality of life, psychological well-being, loneliness, functional ability and use of space at time 1 (study baseline) and time 2 (at 3–4 weeks after the first visit); (b) ethnographic observations in communal spaces of up to 10 care homes to understand how outbreaks and related restrictions to the use of space and social activities impact residents’ well-being. The study will interview only care home residents who have the mental capacity to consent. Data will be compared and integrated to gain a more comprehensive understanding of the impact of outbreaks on residents’ quality of life and well-being.

**Ethics and dissemination:**

The VIVALDI ASCOT and Ethnography Study obtained ethical approval from the Health Research Authority (HRA) Social Care REC (24/IEC08/0001). Only residents with the capacity to consent will be included in the study. Findings will be published in scientific journals.

**STRENGTHS AND LIMITATIONS OF THIS STUDY**

- This study uses a comprehensive mixed-methods design for assessing the impact of respiratory disease outbreaks and related non-pharmaceutical interventions (NPIs) on the quality of life and psychosocial well-being of older residents in care homes, using well-established measures of social care-related quality of life, functional capabilities, loneliness and psychosocial well-being.
- The study includes an innovative application of short-term ethnography to understand the impact of NPIs on the (re)organisation of spaces, activities and communication in care homes following a respiratory outbreak.
- Reliance on notification of outbreaks in care homes may delay data collection.
- In line with ethics requirements, our study only includes participants who can consent to the study, thereby excluding a significant portion of the care home population who may lack the capacity to consent and would need a consultee to advise on their participation.

## Introduction

### Context

 Older residents (aged ≥65) in care homes experienced the highest rates of mortality from SARS-CoV-2 worldwide.^1^,^4^ Outbreaks of other respiratory illnesses, such as influenza, are also common in care homes and represent a major cause of hospitalisation, morbidity and death among residents.^5^,^7^ COVID-19 and other respiratory illnesses can also circulate simultaneously in care homes. Overall, there is an urgent need for evidence to inform proportionate policy on the use of non-pharmaceutical interventions (NPIs), for example, quarantine, social distancing and personal protective equipment, to control the spread of respiratory infections in care homes, which do not compromise residents’ quality of life and well-being.

### Current knowledge

In England, there are about 11 000 care homes that provide accommodation to more than 370 000 older adults.^8^ The resident population of care homes comprises long-term care residents and patients admitted from hospitals. With an average age in the mid-80s, most residents have multiple comorbidities, and two-thirds live with dementia,^6 7^ making them more vulnerable to COVID-19 or other respiratory infections.^1 5^ During the first wave of the COVID-19 pandemic, care home residents in England experienced high rates of mortality from SARS-CoV-2,^9 10^ and residents with dementia were disproportionately affected.^11^ COVID-19 infection control measures, such as self-isolation, restrictions on visits and cancellations of communal activities, were logistically challenging to implement in care homes^12^,^14^ and even high-quality nursing homes with modern built environments experienced substantial numbers of COVID-19 cases and deaths during waves 1 and 2 of the pandemic.^15 16^

To contain and prevent the spread of COVID-19 infection, residents were subjected to strict and prolonged infection control measures (eg, lockdowns, isolation, masking by staff, visits and activity restrictions), which heavily impacted their daily lives and well-being.^17 18^ This led to calls for strategies to find a balance between the physical safety and quality of life of residents, but further evidence is still needed to understand the broader impact of these strategies.^19 20^

Research has shown that COVID-19 infection control measures for care homes were challenging to implement. Care homes are communal living settings, and some residents also share a bedroom and bathroom, which makes measures like isolation and quarantine more challenging to implement. The process of care in these settings requires ‘high touch’ activities, such as bathing and dressing, which makes physical distancing impractical. Complying with public health measures, such as maintaining social distancing and wearing masks, can prove burdensome in these settings, particularly for residents living with cognitive impairment.^21 22^ Moreover, non-verbal communication and physical contact represent important ways of socialising and expressing affection, especially for people with dementia.^23^,^25^

Care homes are residents’ homes, and group activities (eg, dining together), as well as visits from family and friends, are important parts of residents’ daily socialisation. Stringent lockdown measures challenged the aim of care homes as homely environments^14^ and represented a social and functional burden for residents.^26^,^28^ The loneliness and social isolation resulting from outbreak measures^27^ have been associated with poor mental health, depression and anxiety in this population.^28^,^31^ However, the extent of the negative impact of COVID-19 infection control measures on residents’ quality of life and well-being has not been assessed to date, and better evidence is needed to evaluate trade-offs in future policy responses. To date, there remains a paucity of evidence collected directly from care home residents to understand their experiences and preferences related to the impact of respiratory outbreaks and NPIs, as highlighted by the chief medical officer in the technical report on the COVID-19 pandemic.^19^ A standardised approach to describing this impact is also needed to support the development of proportionate, evidence-based policy on the use of NPIs in this setting.^19^ Thus, we propose a multidisciplinary approach to investigating the impact of respiratory disease outbreaks and related NPIs on residents’ quality of life and psychological well-being. Using available measures on mental and functional capabilities, loneliness and social care-related quality of life (SCRQoL), as well as ethnographic investigation, this multidisciplinary approach pragmatically addresses the lack of minimum data available for care home residents and informs future policy.

### Aims of the study

The VIVALDI ASCOT and Ethnography Study aims to investigate the impact of COVID-19 and other respiratory disease outbreaks on residents’ quality of life, collecting data from residents themselves, including those with a degree of cognitive impairment but with the capacity to consent to the study. The study has two primary objectives and one secondary objective. The primary objectives are: (1) to assess the longitudinal impact of COVID-19 or other infection outbreaks on care home residents’ quality of life, psychological well-being, loneliness and functional ability (2) to explore how residents’ use of communal spaces, communication and social activities are affected by outbreaks and how this, in turn, affects their well-being and social connectedness.

A secondary objective is to demonstrate a practical approach to collecting primary data from older residents in care homes, using established measures designed to be administered in adult social care settings (eg, Adult Social Care Outcomes Toolkit (ASCOT)) and immersive methods like short-term ethnography. Ethnographic observations do not rely solely on verbal communication and recall and are better suited to include older residents with diverse communication needs and styles.

We hypothesise that residents in care homes with recent outbreaks will experience worse quality of life, psychological well-being, increased loneliness and impairment in functional ability compared with residents in care homes that did not experience a recent outbreak. In addition, we hypothesise that residents in care homes with recent outbreaks will not use communal spaces, will communicate and engage less in social activities compared with their counterparts in care homes without recent outbreaks.

## Methods and analysis

### Study design

**Figure 1:**
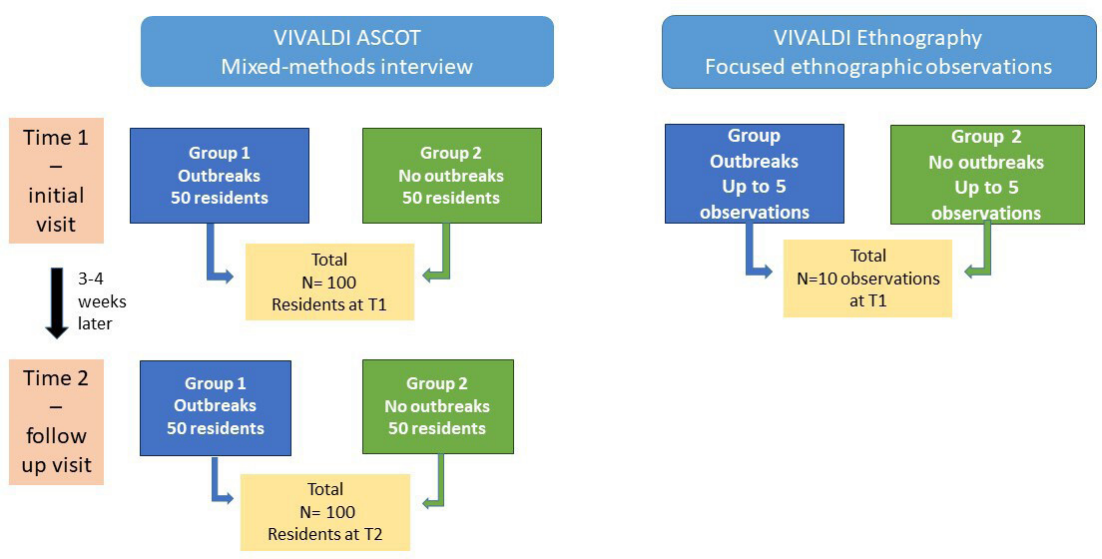
Conceptual flowchart of the VIVALDI ASCOT and Ethnography Study.

VIVALDI ASCOT and Ethnography is a mixed-methods case–control study comprised of two components, which will be running in parallel—as shown in figure 1. The first component (VIVALDI ASCOT) is a mixed-design longitudinal interview comprising the ASCOT survey^32^ and additional measures of mental and functional capability, psychological well-being, social interaction and loneliness as well as qualitative questions on the use of space and communication related to the outbreak measures. We will employ an independent sample design with two groups, namely group 1 (exposed care homes with a recent outbreak) and group 2 (non-exposed care homes without a recent outbreak). By ‘recent’, we mean within 3 weeks of the outbreak being reported by the care home to the local Health Protection team. We will assess the impact of outbreaks on residents’ quality of life, psychological well-being, loneliness, functional ability and use of space at time 1 (study baseline) and time 2 (at 3–4 weeks after the first visit).

The second component (VIVALDI Ethnography) is a short-term cross-sectional ethnographic observation in the communal spaces of care homes in groups 1 and 2 at time 1 to gain a richer understanding of residents’ quality of life and well-being during and in the absence of outbreaks. The ethnographic component will (a) gather in-depth information that cannot be captured or assessed through standardised measures, (b) produce rich data on the context and residents’ experiences that will be used to enrich the findings from the VIVALDI ASCOT interview. Findings will be integrated at the analysis stage. Data from the qualitative questions and ethnographic observations will be compared with the results from the survey questions to aid a more comprehensive picture.

### Patient and public involvement

We conducted a consultation with a group of four patient public involvement (PPI) advisors in Sussex coordinated by Sussex Partnership Foundation NHS Trust. The PPI group was consulted on the following aspects: (a) the suitability of the written materials to recruit residents (eg, invitation letter, participant information sheet); (b) procedures for administering the resident’s subjective well-being and cognitive measures; (c) the choice of measures included in the resident interview. The PPI advisors also reviewed the wording of participant-facing documents to check that the language we are using is suitable, given that the topic of COVID-19 is worrying for many people. This consultation allowed us to make several changes that were beneficial for the study. A lay summary of our study findings will also be developed with our PPI group and disseminated to study participants.

### Study setting

The study will take place in care homes for older adults (65+ years) in the Southeast of England. Ethnographic observations will take place in the communal areas of the care homes included in the study.

### Sample size

#### VIVALDI ASCOT

We aim to interview 100 care home residents who are able to consent to take part in the study. Half of these respondents (n=50) will be recruited from care homes that have had a recent COVID or other respiratory infection outbreak (group 1—exposed), and half of them (n=50) will be recruited from care homes that have not experienced a recent outbreak (group 2—non-exposed). The UKHSA health protection team will alert the research team of any new outbreak in care homes in the southeast areas of Kent, Surrey and Sussex. All respondents will be monitored longitudinally after a 3–4 week period at time 2 (T2).

##### Sample size calculation

A G-Power calculation for an independent sample t-test was conducted to achieve an effect size of 0.8 for the lack of social engagement associated with COVID-19 outbreaks on residents’ quality of life^33 34^ and a power of 0.9, suggesting 34 participants per group. However, given the high probability of dropout for this population group (30%–40%).^35^ We intend to collect 50 participants for each of the two groups, for a total of 100 participants.

Given the potential recruitment challenges for group 1 (exposed) due to the current low incidence of COVID-19 outbreaks in care homes, we have devised a contingency plan to proceed with data collection exclusively for group 2 (non-exposed), thereby adopting an observational study design. A secondary power analysis was conducted for this alternative approach, assuming a multiple linear regression model with five predictors to detect an effect size of 0.233^36^ with a power of 0.8. The power analysis indicated that a total sample size of 61 participants would be required.

##### Inclusion and exclusion criteria

To take part in the VIVALDI ASCOT component of the study, care home residents must be aged 65 and older at the start of the study, live in a care home in the Southeast of England, and have the capacity to consent to the study. The study will only include care home residents who are fluent in the English language.

### VIVALDI Ethnography

We will conduct observations in up to five care homes from group 1 and up to 5 care homes from group 2. No population sampling will be considered for the ethnographic observations. However, we aim to conduct observations when a minimum of 4–5 residents are present in a communal area.

### Recruitment and consent

In this study, particular care and attention need to be paid to issues surrounding residents’ vulnerability, recruitment and informed consent. Thus, it is paramount to have a rigorous and detailed recruitment plan and procedures in place to support residents in making informed decisions. Recruitment will occur at two levels: care homes and residents. The recruitment process began in mid-April 2024, with the goal of completing data collection by May 2025.

#### Care homes

Care homes located in the Upper-tier local authorities of Kent, Surrey, and Sussex will be invited by the National Institute for Health and Care Research (NIHR) Clinical Research Network (CRN) Kent, Surrey, Sussex or directly by the research team to take part in the study. We will also receive alerts of eligible care homes with an outbreak infection (group 1—exposed) through the local Health Protection Team in UKHSA. Care home managers will be invited to agree to the care home participation in the study and provided with a Participant Information Sheet and Consent form. They will have the option to take part in both study components (VIVALDI ASCOT and VIVALDI Ethnography) or only in the VIVALDI ASCOT.

We have collaborated with the NIHR Clinical Research Network and the University of Sussex Research Development Office to develop the Schedule of Events Cost Attribution Template (SoECAT) for the research-associated costs with contributory care homes. Each participating care home will receive a financial contribution to compensate for the time and effort of their staff in supporting the research team with the identification and recruitment of residents.

#### VIVALDI ASCOT

**Figure 2:**
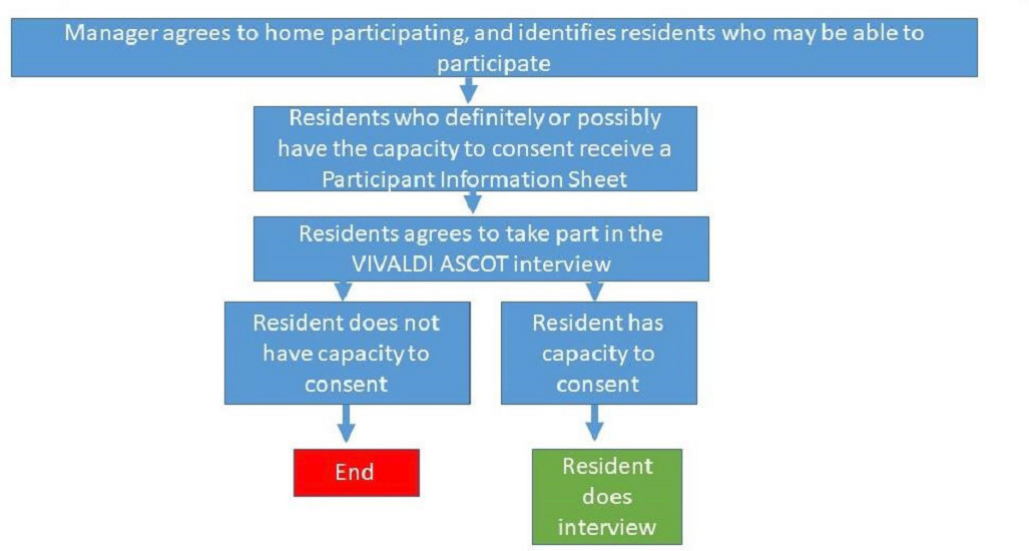
Participation and consent flowchart for the VIVALDI ASCOT component of the study.

Once the care home manager has agreed to take part, the research team will circulate participant information sheets to residents and will be available to answer any questions and clarifications. They will be asked to sign a consent form at the beginning of the T1 and T2 interviews if they wish to participate. A flowchart of the consent process is shown in figure 2. In line with the Mental Capacity Act (MCA),^37^ the capacity to consent will be assumed, but the researchers will carefully follow the MCA Code of Practice^37^ to determine whether the resident has the mental capacity to consent to our study. Only residents who can consent will be included in the study.

#### VIVALDI Ethnography

**Figure 3:**
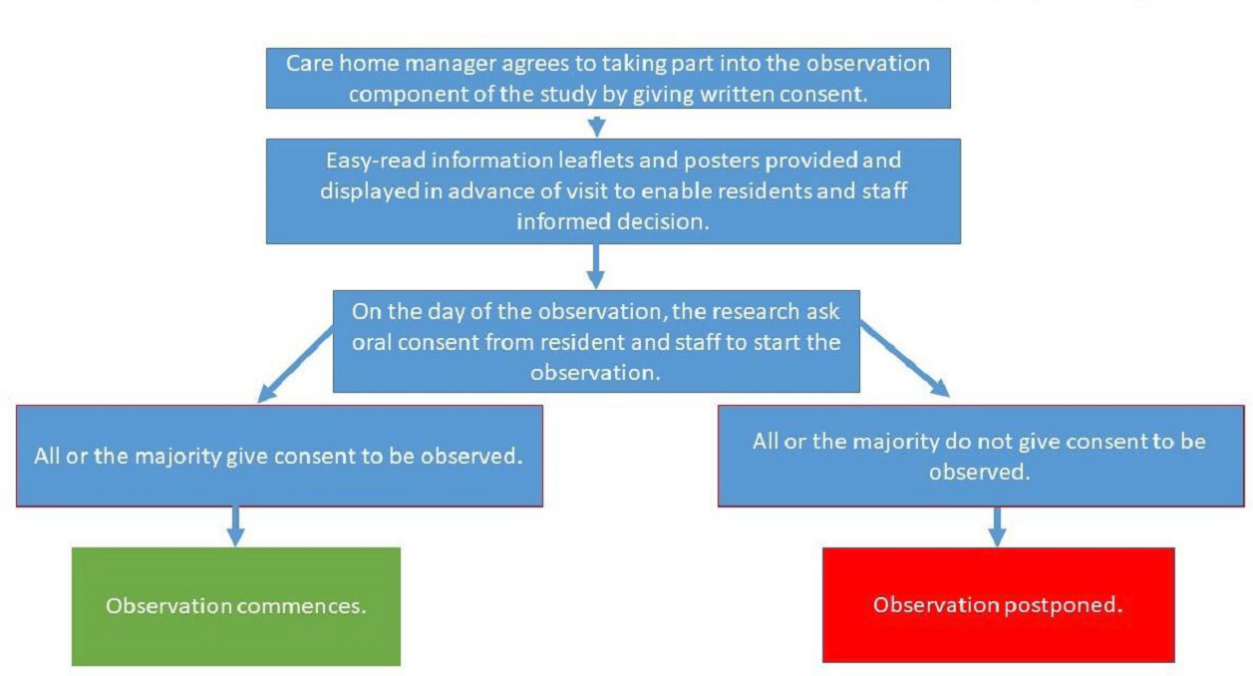
Participation and consent flowchart for the VIVALDI Ethnography component of the study.

Participant information sheets will be circulated to care home residents and staff, and posters will be displayed a few days before the study to inform them about the observation. Residents and staff will be given time to ask questions and clarifications to make an informed decision. On the day of the observation, the researcher will request permission from the residents and staff (and visitors if present) in the communal area to start the observation, and posters will be displayed informing them that the observation is taking place. A flowchart of the consent process is shown in figure 3.

#### Support for residents

We will provide simplified information sheets, detailed explanations and visual aids, which will allow sufficient time for residents to ask questions and make informed decisions for both aspects of the study. This will improve our ability to support all participants in understanding the research information and the nature of our study. Informed consent will be treated as a separate process, and the researchers will be mindful of verbal and non-verbal signs of discomfort, annoyance and mood changes from the residents (and staff) who would indicate that the participant wants to stop their participation.

### Data collection

#### VIVALDI ASCOT

We will undertake all resident interviews in each care home over several days. The study researchers will input the answers to quantitative survey questions using a tablet or laptop on the secure web application REDCap. Interviews will be audio-recorded in their entirety using a voice recording to capture any additional comments that residents make, which may give a richer understanding of their quality of life and psychological well-being. We will use Show Cards, which include survey questions and the choice of responses in a very large font, which is easier to read and provides visual input.

#### VIVALDI Ethnography

Short observations (60 min) will be carried out by a researcher who will take notes on a notepad or tablet, using a mix of observation schedules and personal notes to ensure consistency across observations while also allowing for contingent and contextual events and personal notes to be captured. We will not video or audio record nor take photos during an ethnographic observation. Observations will focus on how the communal space is organised and used, the type of activities taking place and communications between residents and between residents and staff (and visitors if present).

#### Respondent interview measures

The measures assessed in the VIVALDI ASCOT interview are outlined below.

##### Demographics

The interview will start by asking about simple demographic measures such as age, sex, marital status, education and length of stay in residential care.

##### Health conditions

The residents will be asked about their existing health conditions and comorbidities, including whether they had been infected with COVID-19 or suffered from long-term COVID-19. They will also be asked about a number of specific chronic conditions (eg, diabetes, hypertension, etc.).

##### Quality of life—ASCOT

The ASCOT is a well-established set of tools designed to measure SCRQoL outcomes in adult social care settings.^38^ Using the self-report version of the face-to-face Interview Schedule (INT4 Resident), we will evaluate the respondent’s current SCRQoL. The ASCOT-INT4 questions ask respondents to think about their lives and experiences in residential homes across eight distinct domains (see online supplemental material). In agreement with the ASCOT team, we also included a question for each of the three health-related domains: anxiety, low mood and pain. ASCOT is a preference-weighted measure of QoL. This means that raw scores (0, 1, 2, 3) for each domain are converted into the preference-weighted values. These are then added together and entered into a formula to provide an overall quality of life score.

##### Mental and functional capability

We will administer a brief cognitive and functional assessment. The measures employed are described below.

###### Self-reported memory

Participants will be asked to rate their memory in the past 2 months. The answers will consist of: 1. excellent; 2. very good; 3. good; 4. fair and 5. poor.

###### The Montreal Cognitive Assessment (MoCA)

MoCA^39 40^ is a widely used screening tool designed to assess various cognitive functions, including attention, memory, language, visuospatial skills, executive function and orientation. The maximum score is 30. A score of 26 or above is generally considered normal, while a score of 25 or below may indicate some degree of cognitive impairment.

### Functional capability

Activities of Daily Living^41^ refers to activities oriented towards taking care of one’s own body and include the fundamental skills typically needed to manage basic physical needs, such as bathing, dressing, transfer, toileting, feeding and continence.

### Psychological well-being

#### Depressive symptoms

We will measure depressive symptoms with the 8-item version of the Center for Epidemiologic Studies Depression Scale (CES-D),^42 43^ a widely used self-report screening tool used to assess depressive symptoms in the older population.^44^ Each item refers to a specific symptom (eg, depression, happiness, loneliness) and has a yes/no response. Each answer is assigned one point for a maximum score of 8. A total CES-D score of 4 or greater denotes depression.

#### Social interaction and loneliness

Loneliness will be measured using the UCLA 3-Item Loneliness Scale.^45^ We will measure three dimensions of loneliness: relational connectedness, social connectedness and self-perceived isolation, and ask about the level of regular contact with family or friends. Each question has three response options (ie, Hardly ever, Sometimes/Often), which corresponds to a score from 1 to 3. The scores of each answer are added together with a possible total score range of 3 (minimum) to 9 (maximum). The consensus is that the overall scores between 3 and 5 are classified as ‘not lonely’ and the scores between 6 and 9 as ‘lonely’.

#### Use of space and communication

We will ask nine open-ended questions related to the residents’ use of space and activities. These questions are formulated to produce a deeper understanding of residents’ everyday life in the care home, their sense of well-being and ‘homely’ environment, and if and how these are affected by infection control measures. We will ask residents where they like to spend their time (eg, in communal areas, gardens, their room) and how (eg, social activities), and if any of these changes follow an outbreak. These are open-ended questions to understand residents’ experiences, and they do not measure specific outcomes.

### Data analysis

#### Quantitative data

We will conduct an independent t-test between the two independent groups (exposed group vs not exposed group) to analyse quantitative data collected through interviews on SCRQoL, mental and functional capability, psychological well-being, social interaction and loneliness. Subsequently, regression analyses will be conducted to investigate the impact of COVID-19 or any other respiratory infection outbreaks on residents’ quality of life functional and psychosocial outcomes in a cross-sectional design at baseline between the two groups (exposed vs not exposed) while controlling for a wide range of covariates such as age, sex, educational background, type of care home, the season of testing. We will conduct mixed-effect regression models with a random intercept term defined at the level of each home for each specific outcome.

#### Qualitative data

Qualitative data from open-ended questions and ethnographic observation notes will be transcribed verbatim and analysed in the qualitative data analysis software NVivo using thematic analysis.^46^ The analysis will be both deductive, with a focus on the use of space, social activities and connectedness, and communication and inductive, where novel codes and themes will emerge from the data.

#### Data integration

The results and findings from both quantitative and qualitative methods will be compared, contrasted and integrated to produce a more comprehensive understanding of the impact of respiratory outbreaks in care homes.

### Data management and confidentiality

Researchers will comply with the UK’s General Data Protection Regulation and Data Protection Act 2018 requirements regarding the collection, storage, processing and disclosure of personal information.

### Data sharing

At the end of the study, selected anonymised data and metadata will be submitted to the data repository FigShare.

### Risks and risk mitigation

We have identified the following main risks related to this study^1^: inability to find care homes with current outbreaks in a timeframe outside the pandemic when most residents and care workers are currently vaccinated^2^; in case we have care homes with current COVID outbreaks, researchers collecting the data risk getting infected with COVID-19 or other respiratory diseases^3^; inappropriate assessment of capacity to consent^3^; safeguarding disclosure of harm of neglect. The following mitigation strategies are in place for each risk, respectively^1^: an alternative study design and research strategy have been considered as a contingency plan in case we do not achieve the planned sample of care home residents from care homes with current outbreaks (group 1—exposed),^2^ Researchers working in the study are fully vaccinated. They will undertake infection control training prior to data collection and will follow the care home’s policy^3^; researchers have completed Good Clinical Practice and NIHR online training on informed consent with Adults Lacking Capacity^4^; researchers will follow the University of Sussex Safeguarding guidance and Safeguarding referral pathway for work happening outside campus.

## Ethics and dissemination

The study has received ethical approval from the Health Research Authority (HRA) Social Care REC (REC number 24/IEC08/0001).

The study was presented at the UK Health Security Agency (UKHSA) Conference in 2023. Prior to data collection, informed consent will be sought from care home managers and residents. Residents without the mental capacity to consent will not be interviewed. Every resident will receive a £20 voucher as a token of appreciation for their contribution. Findings from the study will be published in scientific journals, and a lay summary will be disseminated to the study participants who expressed interest.

## Discussion

The VIVALDI ASCOT and Ethnography Study represents an innovative and pragmatic approach to producing evidence on the impact of COVID-19 and other respiratory outbreaks on the quality of life and psychological well-being of residents in care homes. The design of the study draws on quantitative, qualitative and ethnographic methods to address important gaps related to the quality and equity of current evidence on outbreak measures in care homes. We contend that evidence is needed to understand the impact of outbreaks on residents’ quality of life and well-being, including residents with cognitive impairment and dementia, who are particularly vulnerable to respiratory outbreaks and whose voices are rarely included in research on infection control measures.

First, by using existing measures of social-care-related quality of life, functional capability and psychosocial well-being, this study proposes a standardised approach to assessing the impact of outbreaks on residents’ quality of life and psychological well-being and informing policy, which is currently lacking. This is an important endeavour as policymaking has primarily focused on clinical and public health priorities, and issues related to NPIs and quality of life in care homes have not been prioritised. To our knowledge, this is the first application of ASCOT to assess the impact of outbreaks on care home residents’ quality of life.

Second, by adopting a short-term ethnography approach^47^ and integrating it with quantitative measurements of SCRQoL, psychological well-being, mental and functional capacity, this study will produce a deeper understanding of how outbreaks impact residents’ well-being and social connectedness. Ethnographic research has shown how the organisation of space and tasks related to hygiene and infection control in care homes affect residents’ dignity and well-being.^48^ This is a promising yet unexplored area of investigation to produce evidence on the impact of outbreaks and NPIs on care home residents’ well-being.

Finally, this study will produce evidence by engaging residents directly to inform policy that has an impact on their lives. Given the age profile and possibility of some of the care home residents experiencing cognitive impairment, as well as the potential sensitivity of the study topic, it is paramount to attend to ethical and logistical considerations and procedures to support as many residents as possible to take part in this study. Our approach to consent is in accordance with the MCA,^37^ and particular attention has been paid to the clarity and accessibility of information in participants’ documents (eg, participant information sheets, etc). The multidisciplinary approach will also aid the inclusion of a broader range of residents’ experiences. The use of qualitative, open-ended questions fashioned in a more conversational way and ethnographic observations will allow the involvement of those residents who may find survey-like questions challenging to respond to.

We wish to highlight several strengths and limitations. A key strength is our methodological rigour and ethical compliance, as approved by the HRA Social Care REC. However, there are limitations regarding the timing of data collection outside the pandemic period and the inclusion criteria, which can only include residents with the mental capacity to consent in compliance with our ethical approval requirements. Given that 60%–70% of care home residents have cognitive impairments or dementia, this ethical consideration might conflict with the newly developed NIHR initiative on Enabling Research in Care Homes. The inclusion of multiple care homes with a recent outbreak is necessary for this study. Still, it could result in delays in data collection and enhance recall bias, as it is always difficult to predict when an outbreak will occur. However, the collaboration with the UKHSA Health Protection Team ensures that the research team receives timely alerts when an outbreak occurs in eligible care homes and that data collection is not overly delayed. Another potential limitation of this study is the utilisation of SCRQoL measures that are not explicitly designed to assess the impact of outbreak measures on resident’s quality of life. ASCOT was intended to measure the impact of social care services on a person’s quality of life. However, based on a preliminary investigation, we undertook to pilot this approach in the context of this study, we concluded that ASCOT could be used to identify and articulate the impact of some NPIs on residents’ quality of life. We will also use other measures related to functional ability, isolation and psychological well-being to collect a more complete set of data.

We hope that our study could provide a conceptual framework for future research aimed at assessing the impact of respiratory outbreaks on care home residents' quality of life and well-being. This framework considers additional outcomes such as psychological well-being, loneliness, functional ability and use of space by integrating ethnographic observations in communal areas. We draw on health behaviour models such as the health belief model^49 50^ and the theory of planned behaviour^51^ to explore how residents’ perceptions and intentions might potentially influence their responses to outbreaks and related interventions.

Data integration is a crucial component of this framework. It combines quantitative and qualitative data to offer a comprehensive understanding of the proposed outcomes, including observations in care homes with and without outbreaks. This integrative approach aligns with the biopsychosocial model,^52^ which emphasises the interaction between biological, psychological and social factors in health outcomes.

Using this framework, which integrates elements from the socio-ecological model^53^ to consider individual, interpersonal and environmental influences, future studies can systematically assess and understand the impacts of respiratory outbreaks on care home residents, ultimately contributing to improved care strategies and policies.

To conclude, this study will produce an evidence-based approach to articulate the implications of NPIs on residents’ quality of life and well-being, generating a deeper and more rounded understanding of trade-offs to inform future policy and planning for care homes. Furthermore, it will contribute to the current considerations on the impact of NPIs on care home residents.

## supplementary material

10.1136/bmjopen-2024-088685 online supplemental file 1
