# Supplementary material for: VIVALDI ASCOT and Ethnography Study: protocol for a mixed-methods longitudinal study to evaluate the impact of COVID-19 and other respiratory infection outbreaks on care home residents’ quality of life and psychosocial well-being
Source: BMJ Open. 2024 Aug 7;14(8):e088685. doi: 10.1136/bmjopen-2024-088685 (PMC11404191; doi:10.1136/bmjopen-2024-088685)
Supplement: online supplemental file 1 [file bmjopen-14-8-s001.pdf]

Supplementary material

ASCOT (INT4 Resident) Tool

Now I would like to know about your experience of living in this care home and how you feel about your quality of life here. I will do this by asking about different aspects of your life.

There are no wrong or right answers. I am just interested in your views and experiences.

If I ask you something that isn't clear, just ask me to explain, and if I ask you a question that you would rather not answer, that is fine; tell me you'd rather not talk about that, and we can move on to another topic.

<u>Control over daily life</u>

1. Which of the following statements best describes how much control you have over your daily life?

(Interviewer prompt: By 'control over daily life', we mean having the choice to do things or have things done for you as you like and when you want. If needed, please prompt: When answering the question, think about your situation at the moment.)

Please tick (□) one box

- □ I have as much control over my daily life as I want
- □ I have adequate control over my daily life
- □ I have some control over my daily life but not enough
- □ I have no control over my daily life

<u>Personal cleanliness and comfort</u>

2. *Thinking about keeping clean and presentable in appearance, which of the following statements best describes your situation?*

(Interviewer prompt: If needed, please prompt: When answering the question, think about your situation at the moment.)

Please tick (□) one box

- □ I feel clean and can present myself the way I like
- □ I feel adequately clean and presentable
- □ I feel less than adequately clean or presentable

- ☐ I don't feel at all clean or presentable

<u>Food and drink</u>

3. Thinking about the food and drink you get, which of the following statements best describes your situation?

(Interviewer prompt: If needed, please prompt: When answering the question, think about your situation at the moment.)

Please tick (☐) one box

- ☐ I get all the food and drink I like when I want
- ☐ I get adequate food and drink at OK times
- ☐ I don't always get adequate or timely food and drink
- ☐ I don't always get adequate or timely food and drink, and I think there is a risk to my health

<u>Personal safety</u>

4. Which of the following statements best describes how safe you feel?

(Interviewer prompt: By 'feeling safe', we mean how safe you feel both inside and outside the care home. This includes fear of abuse, falling or other physical harm. If needed, please prompt: When answering the question, think about your situation at the moment.)

Please tick (☐) one box

- ☐ I feel as safe as I want
- ☐ Generally, I feel adequately safe, but not as safe as I would like
- ☐ I feel less than adequately safe
- ☐ I don't feel safe at all

<u>Social participation & involvement</u>

5*. Thinking about how much contact you have with people you like, which of the following statements best describes your social situation?*

(Interviewer prompt: If needed, please prompt: When answering the question, think about your situation at the moment.)

Please tick (☐) one box

- ☐ I have as much social contact as I want with people I like

- □ I have adequate social contact with people
- □ I have some social contact with people, but not enough
- □ I have little social contact with people and feel socially isolated

Occupation

6. *Which of the following statements best describes how you spend your time?*

(Interviewer prompt: When you are thinking about how you spend your time, please include anything you value or enjoy, such as leisure activities. If needed, please prompt: When answering the question, think about your situation at the moment.)

Please tick (□) one box

- □ I'm able to spend my time as I want, doing things I value or enjoy
- □ I'm able to do enough of the things I value or enjoy with my time
- □ I do some of the things I value or enjoy with my time, but not enough
- □ I don't do anything I value or enjoy with my time

Accommodation, cleanliness, and comfort

7. *Which of the following statements best describes how clean and comfortable your home is?*

(If needed, please prompt: When answering the question, think about your situation at the moment.)

Please tick (□) one box

- □ It is as clean and comfortable as I want here
- □ It is adequately clean and comfortable here
- □ It is not quite clean or comfortable enough here
- □ It is not at all clean or comfortable here

Dignity

8. Which of these statements best describes how having help to do things makes you think and feel about yourself?

Please tick (□) one box

- □ Having help makes me think and feel better about myself

- □ Having help does not affect the way I think or feel about myself

- □ Having help sometimes undermines the way I think or feel about myself

- ☐ Having help completely undermines the way I think or feel about myself

9. *Which of these statements best describes how you are helped or treated and how it makes you think and feel about yourself?*

Please tick (☐) one box

- ☐ The way I’m helped and treated makes me think and feel better about myself
- ☐ The way I’m helped and treated does not affect the way I think or feel about myself
- ☐ The way I’m helped and treated sometimes undermines the way I think and feel about myself
- ☐ The way I’m helped and treated completely undermines the way I think and feel about myself

<u>Anxiety</u>

10. Which of the following statements best describes how often you feel worried or anxious?

(Interviewer prompt: If needed, please prompt: When answering the question, think about your situation at the moment.)

Please tick (☐) one box

- ☐ I hardly ever feel worried or anxious
- ☐ I occasionally feel worried or anxious
- ☐ I often feel worried or anxious
- ☐ I constantly feel worried or anxious

<u>Low mood</u>

11. Which of the following statements best describes how often you feel down or have a low mood?

(Interviewer prompt: If needed, please prompt: When answering the question, think about your situation at the moment.)

Please tick (☐) one box

- ☐ I hardly ever feel down or have a low mood
- ☐ I occasionally feel down or have a low mood

- I often feel down or have a low mood
- I constantly feel down or have a low mood

<u>Pain</u>

12. *Which of the following statements best describes how often you are in pain?*

(Interviewer prompt: If you have pain, but it is well managed through medication or other techniques, please answer how often you are in pain with these things in place. If your pain is not well managed, base your answer on that. If needed, please prompt: When answering the question, think about your current situation.)

Please tick () one box

- I am hardly ever in pain
- I am occasionally in pain
- I am often in pain
- I am constantly in pain
