# Supplementary material for: VIVALDI ASCOT and Ethnography Study: protocol for a mixed-methods longitudinal study to evaluate the impact of COVID-19 and other respiratory infection outbreaks on care home residents’ quality of life and psychosocial well-being
Source: BMJ Open. 2024 Aug 7;14(8):e088685. doi: 10.1136/bmjopen-2024-088685 (PMC11404191; doi:10.1136/bmjopen-2024-088685)
Supplement: Supplementary file 2 [file bmjopen-2024-088685.reviewer_comments.pdf]

PEER REVIEW HISTORY

BMJ Open publishes all reviews undertaken for accepted manuscripts. Reviewers are asked to complete a checklist review form (**http://bmjopen.bmj.com/site/about/resources/checklist.pdf**) and are provided with free text boxes to elaborate on their assessment. These free text comments are reproduced below.

ARTICLE DETAILS

**TITLE (PROVISIONAL)**	The VIVALDI ASCOT and Ethnography Study: protocol for a mixed-methods longitudinal study to evaluate the impact of COVID-19 and other respiratory infection outbreaks on care home residents' quality of life and psychosocial wellbeing
**AUTHORS**	Bertini, Lavinia; Schmidt-Renfree, Nicola; Blackstone, James; Stirrup, Oliver; Adams, Natalie; Cullen-Stephenson, Iona; Krutikov, Maria; Leiser, Ruth; Goscé, Lara; Henderson, Catherine; Flowers, Paul; Shallcross, Laura; Cassell, Jackie; Cadar, Dorina

VERSION 1 – REVIEW

**REVIEWER**	Hunter, Peter<br>Alfred Health, Rehabilitation, Aged and Community Care
**REVIEW RETURNED**	03-Jun-2024

**GENERAL COMMENTS**	Worthwhile study in terms of both the primary and secondary outcomes.<br><br>I would suggest the researchers be up front with any publication or dissemination of results that this study does focus on those who can give consent and note the high percentage of people in care homes have an underlying dementia who are therefore excluded. There should be clarity on what actual guidance is used to determine the ability to consent in the setting of cognitive impairment.

**REVIEWER**	CHAUDHARY, NEETU<br>Punjabi University Faculty of Business Studies
**REVIEW RETURNED**	23-Jun-2024

**GENERAL COMMENTS**	Some references from recent studies could be added for significant improvement of the study.<br>Needs to develop a outcome based conceptual framework for future study.

VERSION 1 – AUTHOR RESPONSE

Reviewer: 1

**Reviewer's comment**	**Response**
I would suggest that the researchers be upfront with any publication or dissemination of results. This study does focus on those who can give consent, and it notes that a high	Thank you for this comment. We agree that this is a critical point to highlight in any study's output, and we have included this aspect in the Strengths and Limitations section of the

percentage of people in care homes have underlying dementia and are therefore excluded. There should be clarity on what actual guidance is used to determine the ability to consent in the setting of cognitive impairment.	Abstract (lines 69-72), as presented below.<br>*'• One limitation of this study is the lack of inclusivity and representation of the broader population in care homes. Specifically, our study only includes participants who can give consent, thereby excluding a significant portion of the care home population who have underlying dementia, a condition stipulated by the HRA Ethics Committee.'*<br><br>We have also added a short discussion of such limitations in the discussion (lines 433-438), as presented below.<br>*'We wish to highlight several strengths and limitations. A key strength is our methodological rigour and ethical compliance, as approved by the HRA Social Care REC. However, there are limitations regarding the timing of data collection outside the pandemic period and the inclusion criteria, which can only include residents with the mental capacity to consent in compliance with our ethical approval requirements. Given that 60-70% of care home residents have cognitive impairments or dementia, this ethical consideration might conflict with the newly developed NIHR initiative on Enabling Research in Care Homes (ENRICH).'*<br><br>Thank you once again for raising this critical point. We have clarified the guidance that we will follow (lines 242-245) as presented below.<br>*'In line with the Mental Capacity Act (MCA) (29), the capacity to consent will be assumed, but the researchers will carefully follow the MCA Code of Practice to determine if the resident has the mental capacity to consent to our study. Only residents who can consent will be included in the study.'*

Reviewer:2

Some references from recent studies could be added for significant improvement of the study.	Thank you for your comment. We have updated our manuscript and added the following references to our manuscript to include further recent studies on the impact of COVID-19 on care home residents' quality of life/wellbeing and methodological references:<br><br>1. UK Health Security Agency. Infection prevention and control (IPC) in adult social care: acute respiratory infection (ARI). 2024. Available from: https://www.gov.uk/government/publications/infection-prevention-and-control-in-adult-social-care-acute-respiratory-infection<br>2. Office for National Statistics. Care homes and estimating the self-funding population, England. 2023. Available from: https://www.ons.gov.uk/peoplepopulationandcommunity/healthandsocialcare/socialcare/articles/carehomesandestimatingtheselffundingpopulationengland/2022to2023#:~:text=3.-,Self-funding<br>3. Olumekor M, Stojić A, Kehler T, Polo F. The Impact of COVID-19 on the Quality of Life and Happiness of Care Home Residents in Croatia: A Cross-Sectional Study. Behavioral Sciences. 2022;12(11).<br>4. Rodriguez-Rodriguez V, Rojo-Perez F, Perez de Arenaza Escribano C, Molina-

	Martínez MÁ, Fernandez-Mayoralas G, Sánchez-González D, et al. The Impact of COVID-19 on Nursing Homes: Study Design and Population Description. International Journal of Environmental Research and Public Health. 2022;19(24).<br>5. Angevaare MJ, Joling KJ, Smalbrugge M, Hertogh CMPM, Twisk JWR, van Hout HPJ. The Effects of the 2020 COVID-19 Lockdown on Mood, Behavior, and Social and Cognitive Functioning in Older Long-Term Care Residents. Journal of the American Medical Directors Association. 2022;23(9).<br>6. Huber A, Seifert A. Retrospective feelings of loneliness during the COVID-19 pandemic among residents of long-term care facilities. Aging and Health Research. 2022;2(1).<br>7. Cortés Zamora EB, Mas Romero M, Tabernero Sahuquillo MT, Avendaño Céspedes A, Andrés-Petrel F, Gómez Ballesteros C, et al. Psychological and Functional Impact of COVID-19 in Long-Term Care Facilities: The COVID-A Study. American Journal of Geriatric Psychiatry. 2022;30(4).<br>8. Rajlic G, Sorensen JM, Mithani A. Exploring Post-COVID-19 Functional Outcomes in Residents in Long-Term Care Homes in British Columbia, Canada. Gerontol Geriatr Med. 2024 May 21;10:23337214241245551. doi: 10.1177/23337214241245551. PMID: 38779377; PMCID: PMC11110507.<br>9. Ceppi, L., Di Pierro, R., Romano, D., & Costantini, G. (2024). Biopsychosocial well-being of nursing home residents during the COVID-19 pandemic: A 3-year longitudinal study. Psychology and Aging. Advanced online publication. https://dx.doi.org/10.1037/pag0000834<br>10. Judith M. Mitchell, Bryan J. Kemp, Quality of Life in Assisted Living Homes: A Multidimensional Analysis, The Journals of Gerontology: Series B, Volume 55, Issue 2, 1 March 2000, Pages P117–P127, https://doi.org/10.1093/geronb/55.2.P117.<br>11. Mohsen, S., El-Masry, R., Ali, O.F. et al. Quality of life during COVID-19 pandemic: a community-based study in Dakahlia governorate, Egypt. Glob Health Res Policy **7**, 15, 2022. https://doi.org/10.1186/s41256-022-00246-2.<br>12. Usman A, Lewis S, Hinsliff-Smith K, et al. Measuring health-related quality of life of care home residents, comparison of self-report with staff proxy responses for EQ-5D-5L and HowRu: protocol for assessing proxy reliability in care home outcome testing. BMJ Open 2018;8:e022127. doi:10.1136/ bmjopen-2018-022127.<br>13. Hoben M, Dymchuk E, Corbett K, Devkota R, Shrestha S, Lam J, et al. Factors Associated With the Quality of Life of Nursing Home Residents During the COVID-19 Pandemic: A Cross-Sectional Study. J Am Med Dir Assoc. 2023 Jun 1 ;24(6):876-884.e5. http://www.jamda.com/article/S1525861023003250/fulltext<br>14. Becker MH. The Health Belief Model and Sick Role Behavior. Health Education Monographs. 1974;2(4):409-419. doi:10.1177/109019817400200407<br>15. Green, E.C., Murphy, E.M. and Gryboski, K. (2020). The Health Belief Model. In The Wiley Encyclopedia of Health Psychology (eds K. Sweeny, M.L. Robbins and L.M. Cohen). https://doi.org/10.1002/9781119057840.ch68<br>16. Bosnjak, Michael et al. "The Theory of Planned Behavior: Selected Recent Advances and Applications." Europe's Journal of Psychology vol. 16,3 352-356. 31 Aug. 2020, doi:10.5964/ejop.v16i3.3107<br>17. Laher N, Bocchinfuso S, Chidiac M, Doherty C, Persson A, Warren E. The Biopsychosocial Impact of COVID-19 on Older Adults. Gerontology and Geriatric Medicine. 2021;7. doi:10.1177/23337214211034274.<br>18. Caperon L, Saville F, Ahern S (2022) Developing a socio-ecological model for community engagement in a health programme in an underserved urban area. PLoS ONE 17(9): e0275092. https://doi.org/10.1371/journal. pone.0275092
Needs to develop an outcome-based	Thank you for this important suggestion. In our discussion, we have now included a section on the overall conceptual framework for our research, providing foundational insights into each existing theoretical framework that can be used to support the

conceptual framework for future study.	integration of these models into future studies, see lines (451-466), as presented below:<br><br>*'We hope that our study could provide a conceptual framework for future research aimed at assessing the impact of respiratory outbreaks on care home residents' quality of life and wellbeing. This framework considers additional outcomes such as psychological wellbeing, loneliness, functional ability, and use of space by integrating ethnographic observations in communal areas. We draw on health behaviour models such as the Health Belief Model (49,50) and the Theory of Planned Behaviour (51), to explore how residents' perceptions and intentions might potentially influence their responses to outbreaks and related interventions.*<br>*Data integration is a crucial component of this framework. It combines quantitative and qualitative data to offer a comprehensive understanding of the proposed outcomes, including observations in care homes with and without outbreaks. This integrative approach aligns with the Biopsychosocial Model (52), which emphasises the interaction between biological, psychological, and social factors in health outcomes.*<br>*Using this framework, which integrates elements from the Socio-Ecological Model (53) to consider individual, interpersonal, and environmental influences, future studies can systematically assess and understand the impacts of respiratory outbreaks on care home residents, ultimately contributing to improved care strategies and policies.'*
